# Red cell distribution width: a novel predictive biomarker for stroke risk after transient ischaemic attack

**DOI:** 10.1080/07853890.2022.2059558

**Published:** 2022-04-26

**Authors:** Ke-Hang Xie, Ling-Ling Liu, Yun-Ru Liang, Chu-Yin Su, Hua Li, Run-Ni Liu, Qing-Qing Chen, Jia-Sheng He, Yong-Kun Ruan, Wang-Kai He

**Affiliations:** a Department of Neurology, Zhuhai Hospital of Integrated Traditional Chinese and Western Medicine, Zhuhai, China; b Department of Nephrology, The Fifth Affiliated Hospital of Sun Yat-Sen University, Zhuhai, China; c Reproductive Endocrine Center, Yangjiang Hospital of Traditional Chinese Medicine, Yangjiang, China

**Keywords:** Transient ischaemic attack, stroke, red blood cell distribution width, predictor, biomarker

## Abstract

**Objective:**

Predicting the prognosis of transient ischaemic attack (TIA) is difficult for many frontline clinicians. The purpose of this study was to determine whether subsequent stroke in TIA patients can be predicted via red blood cell distribution width (RDW).

**Material and methods:**

A total of 360 consecutive patients with new-onset TIA in our stroke centre, were enrolled over the period studied. The patients were divided into three groups: 103 TIA patients, 206 ischaemic stroke (IS) patients and 51 patients with haemorrhagic stroke (HS) within 7 days after TIA. Complete blood count, biochemical parameters and brain imaging were performed on all patients.

**Results:**

The mean RDW values of patients with IS and HS after TIA were significantly higher than patients with TIA (13.35 ± 1.59 vs 12.84 ± 1.19, 13.32 ± 1.08 vs 12.84 ± 1.19, respectively, all *p* ≤ .001). In a multivariate model, RDW was independently associated with stroke after TIA (IS: odds ratio (OR) = 2.52, 95% confidence interval (CI) = 1.46–3.35, *p* = .002; HS: OR = 1.511, 95% CI = 1.101–2.074, *p* = .011). Compared to ABCD^2^ scores, the diagnostic power of RDW in the differentiation of patients with IS after TIA was better (area under curve (AUC): 0.731 vs 0.613, *p* = .015). When an RDW cut-off value of 13.95% was accepted for differentiating patients with IS from TIA, the sensitivity and specificity were 73.7% and 74.3%, respectively. However, the AUC for the ability of the RDW to predict HS was 0.653 (95% CI = 0.589–0.716; *p* < .001).

**Conclusions:**

The early determination of RDW is a promising, rapid, easy and inexpensive biomarker to predict the subsequent stroke in TIA patients, especially for IS.

**KEY MESSAGES**
- The most important result of our study is to show that (1) the higher RDW, the earlier the stroke onset and (2) RDW ≥13.95% has a 2.52-fold risk of ischaemic stroke in TIA patients, and RDW ≥12.85% has a 1.51-fold risk of haemorrhagic stroke.
- As an economic and accessible hematological marker, baseline RDW may serve as a useful biomarker for risk stratification in TIA patients.

## Introduction

Up to 20% of patients with acute ischaemic stroke suffered from a previous transient ischaemic attack (TIA) [1]. Early identification of patients at high risk for stroke after TIA and selection of appropriate treatment can reduce the risk of stroke by 80% and improve prognosis [2]. Although several factors have been combined in different scores (such as ABCD^2^ score: age, blood pressure, presence of clinical weakness or speech disturbance, the duration of symptoms, and the presence or absence of diabetes) in order to stratify the risk after a TIA [3]. Recent data suggest that their clinical value is controversial [4,5]. The National Institute for Health and Care Excellence (NICE) guidelines, updated in 2019, no longer recommend clinical classification using scoring systems such as the ABCD^2^ score [6].

Many studies have been devoted to finding factors that predict subsequent strokes in TIA patients, such as B-type natriuretic peptide [7], soluble CD40 ligand [8], N-acetyl aspartate, glutamate, and taurine [9]. However, detection of the above-mentioned biomarkers is time-consuming and expensive, and difficult to monitor dynamically. In addition to improving risk stratification, some researchers suggest that new prognostic markers could further clarify the underlying pathophysiology or timely adjustment of treatment regimens [6,10,11]. Therefore, we need an inexpensive, easy, available, and sensitive laboratory marker that allows us to reliably predict the prognosis after TIA.

Cumulative evidence suggested that elevated red blood cell distribution width (RDW) was an important prognostic biomarker for predicting functional outcomes and mortality in patients with cerebral infarction [12,13]. Recent studies have found that RDW may reflect the underlying inflammatory state and oxidative stress (OS) damage [14], and therefore is related to the incidence, progression and prognosis of stroke [15,16]. The complex pathophysiological mechanisms behind TIA and stroke are similar [6]. Perhaps, RDW can predict subsequent stroke in TIA patients.

For all we know, no studies have explored the relationship between RDW and TIA prognosis. Therefore, we tested the hypothesis that whether TIA and stroke after TIA can be predicted via the RDW.

## Materials and methods

### Study setting and participants

**Figure 1.**
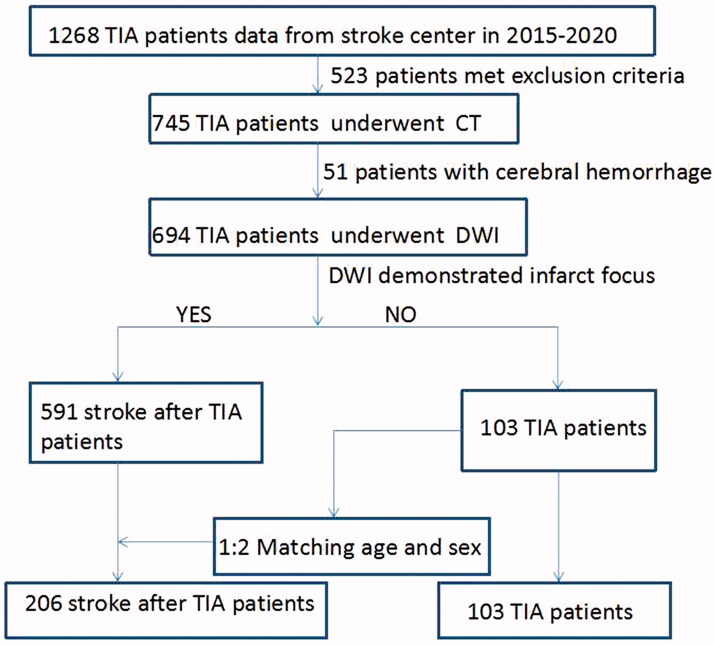
Flow chart for enrollment of the retrospective cohort study.

From January 2015 to December 2020, 360 patients with TIA from our stroke centre were enrolled in this study. All patients were hospitalized and classified into three groups: TIA, ischaemic stroke (IS) and haemorrhagic stroke (HS) after TIA group. A study flow chart is provided in Figure 1.

The inclusion criteria for this study were TIA aged 30–80 years within 48 h of symptom onset. Clinical evaluation, brain CT (computerized tomography) and MRI (magnetic resonance imaging) + MRA (magnetic resonance angiography) + DWI (diffusion-weighted imaging) were performed on each patient. The diagnosis of TIA was based on the clinical features of a presumed vasogenic focal neurological deficit of less than 24 h duration and absence of fresh brain infarction on DWI [17]. IS after TIA was defined as evidence of acute neurological impairment or acute infarction lasting 24 h within 7 days of a TIA based on abnormalities in DWI. Patients with HS were confirmed by CT or MRI.

The etiopathogenic diagnosis of TIA and IS after TIA was classified according to the TOAST (Trial of ORG 10172 in Acute Stroke Treatment) system: large artery atherosclerosis (LAA), cardioembolism (CE), small-vessel occlusion (SVO), a stroke of other undetermined aetiologies (SUE) [18].

### Inclusion and exclusion criteria

Patients were excluded due to: (1) posterior circulation stroke and TIA; (2) history of cardiovascular and cerebrovascular disease, trauma, and surgery within 3 months; (3) other central nervous systematic diseases: epileptic seizure, migraine with aura, peripheral vestibule disease, somatic form disorder, idiopathic facial palsy, transient impaired vision, and brain tumour; (4) severe renal, liver, or heart failure, infection, immunologic diseases, and cancer.

### Clinical and laboratory parameters

Baseline information was collected for all patients, including demographic data, vascular risk factors, time from symptom onset to hospital, blood pressure, ABCD^2^ score, and National Institutes of Health Stroke Scale (NIHSS) score at admission. Clinical outcomes were assessed by MRS (Modified Rankin Scale) after 3 months. Complete blood counts were measured at patients’ arrival at the emergency room using Toshiba, with ethylene diamine tetraacetic acid blood samples. RDW-CV has been extensively studied and calculated according to the following formula: RDW-CV = (standard deviation of red blood cell (RBC) volume/mean RBC volume) × 100 [19]. The normal reference values for RDW in the laboratory are between 11% and 14%. In the present research, we reported the RDW-CV and represented it with RDW.

Serum albumin (ALB), alanine aminotransferase (ALT), uric acid (UA), total bilirubin (TBIL) levels, homocysteine (HCY), creatinine (CR), and fasting blood glucose (FBG) were measured using the Hitachi LST008 analyzer (Hitachi High Technologies, Tokyo, Japan) within the first 12 h after the onset of TIA and after fasting for 8–10 h. Concentrations of total cholesterol (TC), triglyceride (TG), high-density lipoprotein cholesterol (HDL), low-density lipoprotein cholesterol (LDL) and C-reactive protein were measured by the same method. Fibrinogen(FIB) was measured using the Wolfen ACL-TOP-700 automatic coagulation analyzer (Spanish).

### Data collection and outcome assessment

All participants were interviewed using a standardized questionnaire to assess medical histories. Neuroimaging studies were evaluated by two neurologists who were blinded to the clinical data and independently identified TIA and IS or HS after TIA. Disputes were resolved by consensus.

### Statistical analysis

Normally distributed continuous variables were compared by the Student’s *t*-tests and expressed as the mean standard deviations. Continuous variables that were not normally distributed were represented by the inter-quartile range (IQR) and compared by the Mann–Whitney *U* test. Categorical variables were expressed as absolute and relative frequencies and were compared using a chi-square test. Pearson’s method was used for correlation analysis of normal distribution materials, and Spearman’s method was used for correlation analysis of non-normal distribution materials. In order to identify determinants of stroke after TIA, all possible variables with *p* < .05 in univariate analysis were then input into a forward logistic regression model. The results were expressed as adjusted odds ratio (OR) with the corresponding 95% confidence interval (CI). If RDW was an independent risk factor for stroke after TIA, the receiver operating characteristic curve (ROC) for predicting TIA progression with RDW was drawn. When the Youden index was the largest, the optimal diagnostic cut-off point for RDW was calculated, and then the sensitivity and specificity were calculated respectively. We compared ABCD^2^ scores and RDW levels under the ROC curve. *p* < .05 was defined as statistically significant, and all statistical analyses were performed with IBM SPSS statistical version 24 (SPSS Inc. Chicago, IL, USA).

### Ethical considerations

This was a retrospective, cross-sectional study that did not involve clinical or animal studies and would not have had any effect on patient outcomes. According to the statement on ethics approval by the ethics committee composed of our hospital, the requirement for ethics approval was exempted.

## Results

### General characteristics of the subjects

Patients’ baseline characteristics and clinical properties are summarised in Table 1. A total of 360 patients were included in the study: 103 patients with TIA (TIA group), 206 patients with IS (IS group) and 51 HS patients after TIA (HS group). TIA and IS groups accounted for 57% of males with a mean age of 58.94 ± 10.54 years, while the HS group accounted for 68% of males with a mean age of 58.88 ± 1.33 years (all *p* > .05).

**Table 1.** The clinical characteristics of the study samples.

Characteristics	TIA (*n* = 103)	IS (*n* = 206)	HS (*n* = 51)	*p*^1^*-*Value	*p*^2^-Value	*p*^3^-Value
Age (years), mean (SD)	58.94 (10.54)	58.94 (10.54)	58.88 (1.33)		.895	.884
Male, *n* (%)	59 (57)	118 (57)	39 (68)		.173	.142
Alcohol consumption, *n* (%)	19 (17)	42 (20)	9 (18)	.461	.554	.444
Current smoking, *n* (%)	23 (22)	67 (33)	12 (24)	.084	.521	.225
Hypertension, *n* (%)	64 (62)	145 (70)	39 (77)	.157	.258	.409
Diabetes mellitus, *n* (%)	26 (25)	82 (40)	19 (37)	.030*	.171	.476
Coronary heart disease, *n* (%)	11 (11)	28 (14)	5 (10)	.586	.561	.354
Time from onset to hospital (h)	19.52 (8.00)	21.20 (12.00)	24.42 (9.00)	.258	.000***	.000***
BMI, mean (SD)	24.97 (3.44)	25.36 (3.03)	24.42 (2.73)	.155	.485	.035*
SBP (mm Hg), mean (SD)	144.70 (23.46)	155.67 (25.09)	142.59 (20.12)	.000***	.759	.001**
DBP (mm Hg), mean (SD)	89.43 (15.45)	96.22 (14.94)	91.57 (13.02)	.000***	.195	.041*
WBC (10^9^/Ul), mean (SD)	7.20 (2.04)	7.49 (1.95)	7.63 (1.86)	.130	.099	.593
N/L, mean (SD)	2.70 (1.46)	2.72 (1.47)	2.76 (1.38)	.016*	.283	.431
HGB (10^9^/L), mean (SD)	141.06 (13.34)	141.79 (15.66)	143.92 (14.48)	.715	.178	.229
RBC (10^9^/L), mean (SD)	4.68 (0.56)	4.74 (0.47)	4.71 (0.40)	.082	.364	.749
PLT (10^9^/L), mean (SD)	232.56 (54.74)	236.11 (61.30)	208.45 (53.06)	.615	.038*	.009**
ALT (U/L), median (IQR)	19 (14–23)	18 (13–27)	19 (14–25)	.970	.557	.600
TBIL (μmol/L), mean (SD)	13.72 (4.92)	12.31 (5.91)	13.05 (5.35)	.038*	.029*	.574
CR (μmol/L), mean (SD)	70.56 (20.60)	68.66 (17.59)	67.28 (15.71)	.068	.011*	.002**
CRP (mg/L), median (IQR)	2.80 (1.10–4.70)	3.90 (1.70–7.20)	3.50 (1.60–6.70)	.002*	.048*	.711
FIB (g/L), median (IQR)	2.59 (2.18–2.93)	2.57 (2.31–3.12)	2.67 (2.30–3.03)	.111	.207	.945
ALB (g/L), median (IQR)	42.01 (40.0–43.8)	40.80 (39.18–42.65)	41.50 (38.80–43.3)	.001**	.038*	.950
UA (μmol/L), mean (SD)	365.24 (102.49)	344.44 (91.79)	352 (90.27)	.187	.216	.017*
HCY (mmol/L), mean (SD)	10.36 (4.50)	12.28 (6.96)	13.05 (8.19)	.040*	.044*	.589
TG (mmol/L), mean (SD)	1.53 (0.76)	1.65 (0.87)	1.79 (1.41)	.193	.250	.856
TC (mmol/L), mean (SD)	4.98 (1.05)	5.07 (1.17)	4.71 (0.89)	.660	.077	.023*
LDL (mmol/L), mean (SD)	3.04 (0.89)	3.20 (1.03)	2.93 (0.72)	.259	.562	.111
HDL (mmol/L), mean (SD)	1.29 (0.27)	1.24 (0.27)	1.18 (0.20)	.179	.018*	.164
FBG (mmol/L), mean (SD)	6.09 (1.79)	7.22 (2.97)	6.20 (1.95)	.000***	.934	.010*
RDW (%)	12.84 (1.19)	13.35 (1.59)	13.32 (1.08)	.000***	.001**	.255
Medication history, *n* (%)						
Antihypertensive therapy	50 (49)	117 (57)	18 (35)	.184	.138	.077
Antiglycemic therapy	12 (11)	45 (22)	8 (16)	.573	.867	.367
Antiplatelet therapy	36 (35)	94 (46)	26 (51)	.095	.143	.389
Statin therapy	5 (5)	16 (8)	6 (12)	.713	.133	.279
Thrombolysis treatment	29 (28)	92 (45)		.001**		
Stroke scale						
ABCD^2^ scores median (IQR)	5 (4–6)	6 (4–7)	6 (5–7)	.001**	.000***	.253
NIHSS scores median (IQR)	3 (3–5)	6 (3–7)	6 (4–8)	.000***	.000***	.665
MRS scores median (IQR)	0	1 (0–1)	1 (0–2)	.000***	.000***	.614
Aetiology classification, *n* (%)						
LAA	20 (19)	45 (22)		.404		
SVO	28 (27)	89 (43)		.038*		
CE	16 (16)	35 (17)		.459		
SUE	39 (38)	37 (18)		.003**		
Mortality, *n* (%)						
Number of deaths after 30 days	0 (0)	2 (0.009)	1 (0.019)			.489

BMI: body mass index, defined as weight in kilograms divided by the square of height in metres; SBP: systolic blood pressure; DBP: diastolic blood pressure; WBC: white blood cells; N/L:Neutrophilic/lymphocytes; HGB: haemoglobin; RBC: red blood cell; RDW: red blood cell distribution width; PLT: blood platelet; ALT: alanine transaminase; TBIL: total bilirubin；CR: creatinine; CRP: c reactive protein; FIB: fibrinogen; ALB: albumin; UA: uric acid; HCY: Homocysteine; TG: triglycerid; TC: total cholesterol; LDL: low-density lipoprotein cholesterol; HDL: high-density lipoprotein cholesterol; FBG: fasting blood-glucose; ABCD^2^:Age, Blood Pressure, Clinical Features, Duration, and Diabetes; National Institutes of Health Stroke Scale: NIHSS; Modified Rankin Scale: MRS; Values are expressed as Mean ± SD, median (IQR). *p*^1^: TIA vs. IS patients; *p*^2^: TIA patients vs. HS patients; *p*^3^: IS patients vs. HS patients. The differences were considered significant if *p*-value < .05. ***p-Value < .001, **p-value < .01, *p-value < .05.

No differences were found in age, BMI, history of smoking and drinking, hypertension, coronary heart disease, medication history (antihypertensive, antiglycemic, antiplatelet, and statin therapy) in the three groups (all *p* > .05). In contrast, systolic and diastolic blood pressure (*p* = .000) in IS group were significantly highest in the three groups. IS group had more patients with diabetes than TIA group (*p* = .030). Of the three groups, the HS group had the longest time from onset to hospitalization (all *p* = .000). IS patients had better compliance than TIA when it came to thrombolysis treatment (28% vs 45%, *p* = .116). ABCD^2^, NHISS, and MRS scores were significantly higher in IS and HS patients than in TIA patients (all *p <* .05), but there were no differences between IS and HS groups in all three scores (all *p* > .05).

For haematologic and metabolic indicators, serum levels of CRP, TB, ALB and HCY in patients with stroke (both IS and HS group) were lower than those in the TIA group (all *p* < .05). As compared with the TIA and IS group, serum levels of CR in HS were significantly lower (both *p* < .05). N/L and FBG levels were higher in IS than in the TIA group (*p* = .000). When compared with IS, serum levels of UA in HS were higher (*p* < .05). RDW values in IS and HS groups were significantly higher than in the TIA group (13.35% vs 12.84%, *p* = .000; 13.32% vs 12.84%, *p =* .001) (Figure 3(A)).

### Spearman’s or Pearson’s correlation analysis between RDW and inflammatory and OS markers

**Figure 2.**
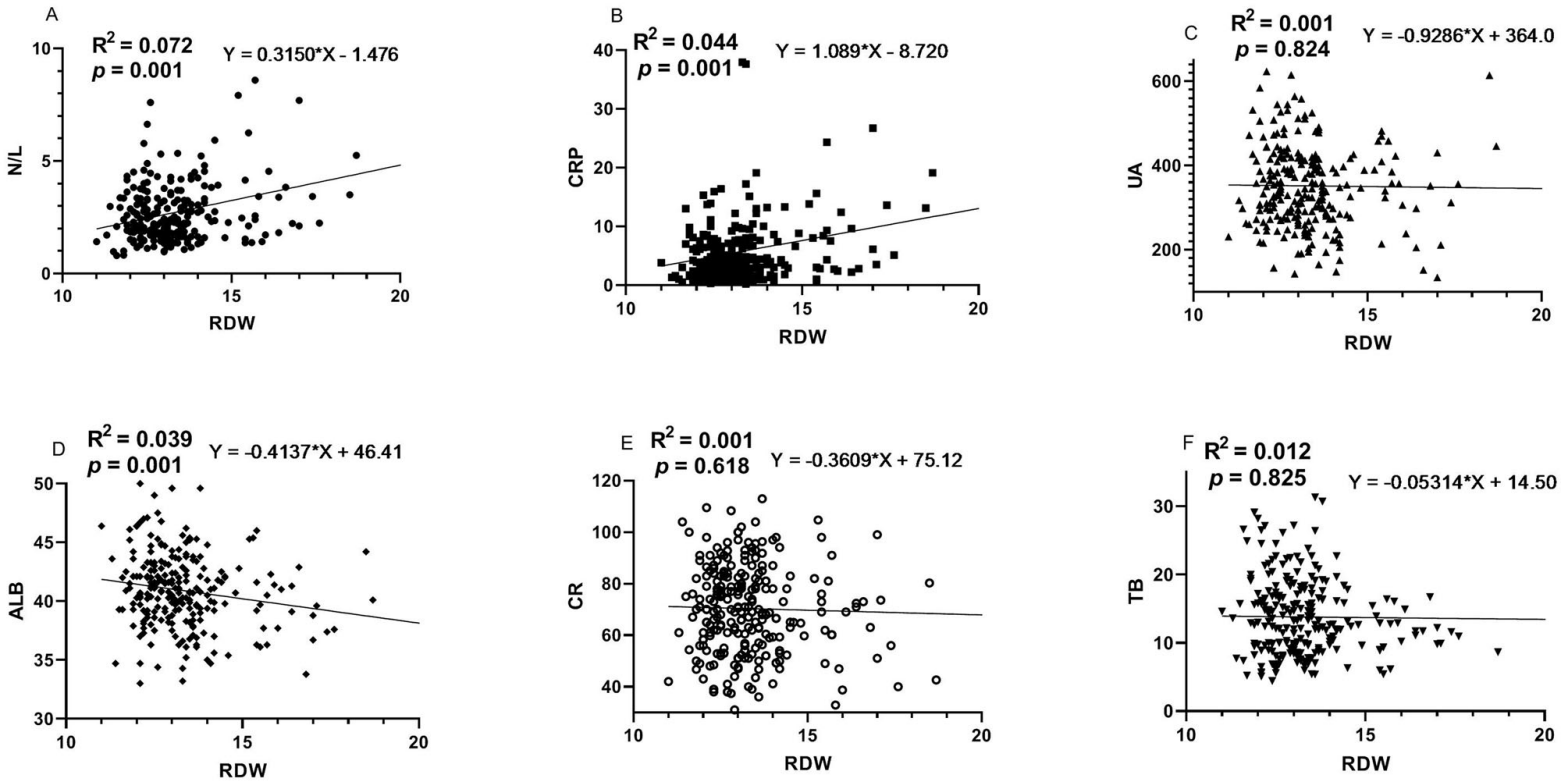
(A–F) Correlation analysis about oxidative stress and inflammatory markers.

Figure 2(A,B) shows the results of the correlation between RDW and inflammatory markers in stroke patients, suggesting RDW was positively correlated with CRP and N/L (both *p* < .05). UA, TB, ALB and CR were considered as antioxidant status biomarkers [20]. RDW was negatively correlated with ALB (*p* < .05). However, RDW had no correlation with UA, TB, and CR (all *p* > .05) (Figure 2(C–F)).

### Multiple logistic regression analysis

Our multivariable regression analysis model suggested that RDW (*p* = .002; OR = 2.52; 95%CI = 1.46–3.35) could independently predict IS in TIA patients, and elevation in serum RDW was associated with HS (*p* = .011; OR = 1.511; 95% CI = 1.101–2.074) (Table 2).

**Table 2.** Risk factors for stroke using multiple logistic regression.

	OR	95% CI	*p-*Value
Risk factors for IS			
RDW	2.523	1.464–3.345	.002**
CRP	1.098	1.035–1.164	.007*
ALB	0.844	0.766–0.930	.001**
HCY	1.062	1.010–1.116	.019*
GLU	1.240	1.062–1.449	.007*
Risk factors for HS			
RDW	1.511	1.101–2.074	.011*
TB	1.079	1.006–1.156	.032*

The differences were considered significant if *p*-value < .05. ***p-Value <.001, **p-value < .01, *p-value < .05.

### Distribution characteristics of RDW value in age, stroke onset time and stroke aetiology

#### RDW increases with age

**Figure 3.**
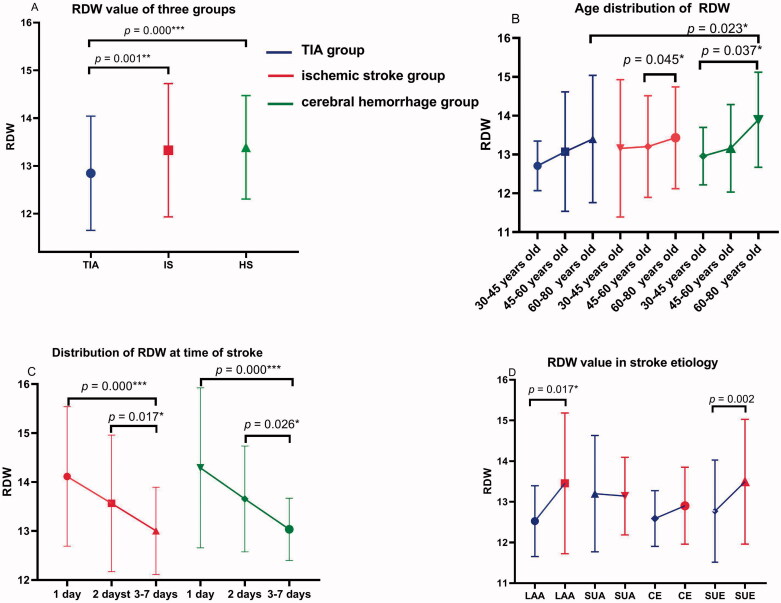
(A–D) Distribution characteristics of RDW value in TIA and stroke group, age, stroke onset time and stroke aetiology.

In terms of age segmentation, our results showed RDW values in the three groups increased with age (although not all *p* were significant) (Figure 3(B)).

#### The higher RDW, the earlier the stroke onset after TIA

By categorizing the time from TIA to stroke, it can be concluded that the higher the baseline RDW, the shorter the stroke onset (all *p* < .05) (Figure 3(C)).

#### Distribution characteristics of RDW value in stroke aetiology

Higher serum levels of RDW in IS group than in the TIA group were observed among LAA and SUE subgroups (*p* = .017 and .002, respectively) (Table 3 and Figure 3(D)). No difference was found in SUA and CE subgroup (*p* > .05).

**Table 3.** Distribution characteristics of RDW value in age, stroke onset time and stroke aetiology.

	TIA (*n* = 103)	IS (*n* = 206)	HS (*n* = 51)	*p*^1^*-*Value	*p*^2^-Value	*p*^3^-Value
Age distribution of RDW						
≥30 to <45 years, median (SD)	12.71 (0.64)	13.16 (1.77)	12.76 (0.79)	.863	.819	.975
≥45 to <60 years, median (SD)	13.07 (1.54)	13.21 (1.31)	13.09 (0.83)	.384	.824	.453
≥60 to ≤80 years, median (SD)	13.26 (0.83)	13.55 (1.79)	13.90 (1.22)	.184	.023*	.064
Time of stroke after TIA						
1 day, median (SD)		14.11 (1.43)	14.29 (1.63)			.749
2 days, median (SD)		13.57 (1.39)	13.66 (1.08)			.390
3–7 days, median (SD)		13.00 (0.89)	13.03 (0.64)			.423
Stroke aetiology						
LAA (SD)	12.53 (0.87)	13.45 (1.73)		.017*		
SVO (SD)	13.20 (1.43)	13.13 (0.95)		.682		
CE (SD)	12.59 (0.68)	12.90 (0.89)		.259		
SUE (SD)	12.77 (1.26)	13.57 (1.99)		.002*		

*p*^1^: TIA vs. IS patients; *p*^2^: TIA patients vs. HS patients; *p*^3^: IS patients vs. HS patients. The differences were considered significant if *p*-value < .05. ***p-Value < .001, **p-value < .01, *p-value < .05.

#### Efficacy of RDW in predicting stroke

**Figure 4.**
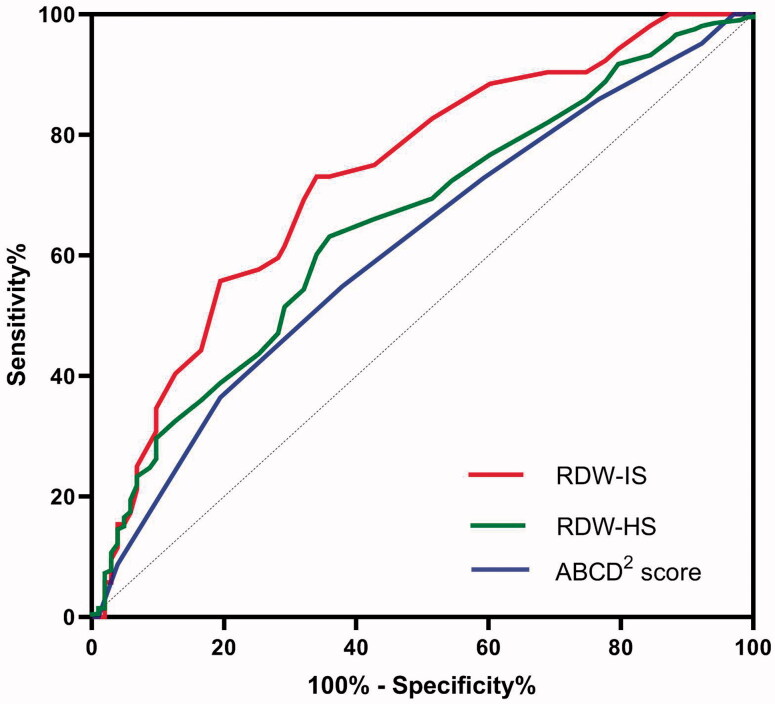
The ROC curve analysis of admission RDW for predicting the stroke after TIA.

The ROC curve analysis of RDW values for predicting the risk of stroke after TIA revealed that RDW can predict IS better than HS after TIA (Figure 4). (1) The area under the ROC curve for IS was 0.731 (95% CI, 0.648–0.814; *p* = .000), and the best predictive RDW value was 13.95% (73.7% sensitivity and 74.3% specificity). Meanwhile, the AUC for ABCD^2^ score was 0.613 (95% CI, 0.547–0.678; *p* = .001). RDW was better than ABCD^2^ score in predicting IS after TIA (*Z* = 2.19, *p* = .015). (2) The AUC for the ability of the RDW to predict HS was 0.653 (95% CI, 0.589–0.716; *p* < .001), and the optimal cut-off value was 12.85%.

#### Efficacy of RDW in predicting aetiology

**Figure 5.**
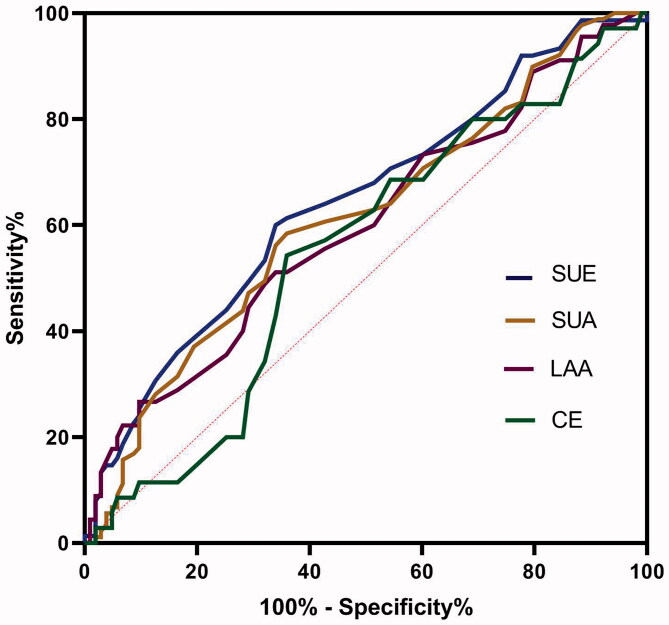
The ROC curve analysis of admission RDW for predicting the stroke aetiology.

To identify stroke aetiology, the AUC for LAA, SUA, CE and SUE were 0.598 (0.496–0.699), 0.612 (0.532–0.692), 0.548 (0.441–0.656) and 0.644 (0.562–0.726), respectively (*p* = .059, .007, .396 and .001, respectively). The cut-off value of SUA and SUE was 12.95% (sensitivity 60%, specificity 66.02%) and 12.86% (sensitivity 58.43%, specificity 64.08%) (Figure 5).

## Discussion

The result of our study showed that RDW was significantly higher in the stroke group than TIA group and elevated RDW was associated with an increased risk of IS and HS in TIA patients. The current study is, so far as we know, the first to clarify the predictive value of elevated baseline RDW levels in patients with TIA.

Previous studies found an association between RDW and incident stroke in the general population, which was independent of anaemia [21]. The higher RDW values measured in stroke patients were associated with adverse functional outcomes and mortality [12]. Moreover, increased RDW has proven to be a potent predictor of neuronal damage [22], haemorrhagic transformation [23], higher mortality after intravenous thrombolysis [24], and atrial fibrillation [25] in IS patients.

The mechanism by which high RDW is associated with stroke progression and poor prognosis is not fully understood. OS and subsequent subclinical inflammation may play important pathophysiological mechanisms for this clinical phenomenon, due to increased RDW comprehensively representing higher levels of OS damage [14,26]. Lorente et al. [27] found that patients with malignant middle cerebral artery infarction (MMACAI) and eventual death showed higher RDW, blood malondialdehyde (MDA) levels, and tumour necrosis factor-α (TNF-α) levels than survivors and these parameters are correlated. They suggested that the association between mortality and RDW in MMACAI patients may be due to higher OS and higher inflammatory status [27]. Sequentially, we hypothesize that patients with TIA are more prone to stroke if accompanied by higher RDW.

As mentioned above, elevated RDW was associated with the potential inflammatory state and OS damage, and may predict the incidence and prognosis of TIA patients. The explanation of the correlation between increased RDW level and stroke after TIA may be as follows.

### First, higher OS status in stroke patients after TIA

Cumulative evidence indicated that serum levels of OS increased with RDW. A 24-month follow-up study of 786 women with moderate and severe disabilities found that their serum oxidant levels increased as RDW increased [28]. And UA, TB, ALB and CR were found to comprehensively reflect the antioxidant status [20]. In this study, we found that the stroke patients had higher RDW than TIA patients. Furthermore, UA, TB, ALB and CR were lower in the stroke group than in the TIA group, and RDW values were inversely correlated with the levels of these four markers (although not all findings were statistically significant, Figure 2(C–F)). These results suggested that stroke patients with higher RDW had lower antioxidant capacity.

Ischaemia and reperfusion injury can induce OS through the production of reactive oxygen species [29]. The TIA animal model suggested that oxygen free radicals produced during reperfusion of ischaemic brain injury might be the main cause of reperfusion injury [30]. OS damage and antioxidant levels have been shown to be associated with neuronal damage/protection during cerebral ischaemic and reperfusion, which play a role in functional outcome and mortality [31]. Thus, the imbalance between antioxidant and oxidant will cause oxidative damage, which can lead to stroke. From a metabolic point of view, anaerobic glucose metabolism and hyperglycolysis after cerebral ischaemia produced lactic acid, exacerbating brain tissue injury through enhancement of free radical formation and mitochondrial failure [32].

Many studies have shown that the incidence of stroke is higher with increasing age [33,34], which also proves from another perspective that the aetiology of a stroke may be the result of long-term OS accumulation [35]. Consistent with previous research [36], our study showed that RDW values in the three groups increased with age (Figure 3(C)). In our study, increased RDW with higher OS was associated with stroke after TIA, suggesting an important role of OS in pathogenesis. The reasons behind this interesting result deserve further investigation.

### Second, higher inflammation status in stroke patients after TIA

The role of inflammation in the ischaemic cascade after TIA is well known. Inflammatory mechanisms are central to the pathogenesis and progression of atherosclerosis, plaque rupture [37], thrombosis [38], and stroke [39]. A rich body of literature demonstrates that inflammation is associated with increased stroke risk and may be an important determinant of outcomes [40]. Inflammatory biomarkers such as P-selectin have been considered to be predictors of stroke after TIA [41].

OS can reduce RBC lifespan, and inflammation is closely related to suppressed RBC production, both of which may increase RDW levels [42,43]. Researchers have found that serum levels of inflammation increased with RDW [28]. A study of 3845 adult outpatient subjects further supported this hypothesis, demonstrating a strong, hierarchical and independent relationship between RDW and hsCRP levels [44]. In addition, some scholars have found that RDW and CRP are positively correlated, which further confirmed the hypothesis that RDW is an inflammation marker [45]. Moreover, another study revealed that older women in the higher quartile of RDW were associated with a higher concentration of interleukin-6, suggesting the predictive value of RDW in serum antoxidants and inflammation [46]. To some extent, our results are consistent with the conclusion of their research. In our study, both the values of RDW, N/L and CRP were significantly higher in stroke patients. Moreover, RDW was positively correlated with CRP and N/L, suggesting that RD patients suffered from a more severe inflammatory response.

In all, our study suggested that patients with stroke had upregulated inflammation, and the OS-related inflammation may be a possible mechanism in the pathogenesis of stroke after TIA.

### Third, microcirculation disturbance in stroke patients after TIA

OS is a critical factor causing microcirculation disturbance [47]. With the increase of RDW, the size of RBC is not uniform, and its deformation causes changes in peripheral blood circulation function. This may be an independent or synergistic factor for increasing circulatory resistance and vascular occlusion [48]. Increased RBC aggregation and reduced deformability were observed in the pathophysiology of circulatory disorders, including myocardial infarction, inflammation, and stroke [49]. These hemorheological parameters interrupt microcirculation through narrow capillaries in ischaemic tissue [50]. The present study revealed that FIB levels were elevated in RD group, indicating higher procoagulant status and worse microcirculation of RD. This was in agreement with a previous study focussed on microcirculation, which found that elevated RDW leads to slow coronary flow.

### RDW and its aetiology

Some scholars found that elevated RDW could lead to poor collateral flow and increased final infarct volume in LAA-related stroke patients [51]. In our stroke subgroup classification, it was found that the LAA subgroup in the IS group had higher RDW values. The RDW was positively correlated with the cholesterol content of the erythrocyte membrane, which increases the volume of the necrotic lipid core and leads to the rupture of atherosclerotic plaque [52]. This may be the reason for the higher RDW value in the LAA group.

More importantly, the comparison of different AUC (Figure 4, IS:0.731 vs 0.613; HS:0.653 vs 0.613) supported that RDW values could improve predictive power when compared with the ABCD^2^ score. To further investigate the necessity of the addition of RDW to a scoring system, larger-sample studies are needed.

In conclusion, our findings found the prognostic value of RDW in patients with TIA. Increased RDW likely reflected the presence of OS, inflammation and poor microcirculation. Based on the above evidence, we look forward to more studies confirming that RDW is a powerful predictor of subsequent stroke in TIA patients.

## Limitations

One limitation of our study was that several markers of inflammation and OS, such as TNF-α and MDA, were not adequately evaluated. Another limitation was that the RDW was measured only once, which may increase the possibility of analyzing defects. Thirdly, the sample of this retrospective study was small, which may lead to biases in the results of the study. Fourth, this study was lack of genetic data that explains the low values of the antioxidant factor. This may be considered in our future study.

## Conclusions

In summary, this study was the first to reveal that elevated RDW was an independent predictor of subsequent stroke in TIA patients. As an economic and accessible hematological marker, baseline RDW may serve as a useful biomarker for risk stratification in TIA patients.

## Ethics approval and consent to participate

This was a retrospective study of secondary utilization of medical records/biological specimens, which would not have had any effect on patient outcomes. The paper’s data were part of patients’ standard care. Any information related to patients’ privacy will not be disclosed to the public, and will not be used for any commercial purposes. There is therefore no risk to the patients. In view of the above reasons, the Ethics Committee of Zhuhai Hospital of Integrated Traditional Chinese and Western Medicine agreed to exempt the informed consent of patients in this project. The ethical batch number is:20211101001.

## Consent for publication

All authors have read and agreed to the published version of the manuscript.

## Author contributions

Conceptualization, Ke-Hang Xie.; Data curation, Ke-Hang Xie.; Formal analysis, Ling-Ling Liu.; Investigation, Yun-Ru Liang.; Project administration, Chu-Yin Su. and Hua Li.; Resources, Qing-Qing Chen.; Software, Run-Ni Liu.; Supervision, Wang-Kai He.; Validation, Yong-Kun Ruan.; Visualisation, Jia-Sheng He.; Writing the original draft, Yun-Ru Liang.; Writing review and editing, Ke-Hang Xie.

## Supplementary Material

Supplemental Material

## Data Availability

The data that support the findings of this study are available on request from the corresponding author. The data are not publicly available due to privacy or ethical restrictions.

## Abbreviations

RDW: red blood cell distribution width
TIA: transient ischaemic attack
IS: ischaemic stroke
HS: haemorrhagic stroke
ABCD2 score: age, blood pressure, the presence of clinical weakness or speech disturbance, the duration of symptoms, and the presence or absence of diabetes
OS: oxidative stress
CT: computerized tomography
MRI: magnetic resonance imaging
MRA: magnetic resonance angiography
DWI: diffusion weighted imaging
RBC: red blood cell
IQR: inter-quartile range
CI: confidence interval
ROC: receiver operating characteristic curve
OR: odds ratio
MDA: malondialdehyde
TNF-α: tumour necrosis factor-α
ROS: reactive oxygen species
RNS: reactive nitrogen species

## References

[1] Rothwell PM, Warlow CP. Timing of TIAs preceding stroke: time window for prevention is very short. Neurology. 2005;64(5):817–820. 15753415 10.1212/01.WNL.0000152985.32732.EE

[2] Rothwell PM, Giles MF, Chandratheva A, et al. Effect of urgent treatment of transient ischaemic attack and minor stroke on early recurrent stroke (EXPRESS study): a prospective population-based sequential comparison. Lancet. 2007;370(9596):1432–1442. 17928046 10.1016/S0140-6736(07)61448-2

[3] Johnston SC, Rothwell PM, Nguyen-Huynh MN, et al. Validation and refinement of scores to predict very early stroke risk after transient ischaemic attack. Lancet. 2007;369(9558):283–292. 17258668 10.1016/S0140-6736(07)60150-0

[4] Amarenco P, Labreuche J, Lavallée PC, et al. Does ABCD2 score below 4 allow more time to evaluate patients with a transient ischemic attack? Stroke. 2009;40(9):3091–3095. 19520988 10.1161/STROKEAHA.109.552042

[5] Amarenco P, Labreuche J, Lavallee PC. Patients with transient ischemic attack with ABCD2 < 4 can have similar 90-day stroke risk as patients with transient ischemic attack with ABCD2 >/=4. Stroke. 2012;43:863–865. 22156685 10.1161/STROKEAHA.111.636506

[6] NIHCE. Stroke and transient ischaemic attack in over 16s: diagnosis and initial management. London (UK); National Institute for Health and Care Excellence; 2019. 31211538

[7] Rodriguez-Castro E, Hervella P, Lopez-Dequidt I, et al. NT-pro-BNP: a novel predictor of stroke risk after transient ischemic attack. Int J Cardiol. 2020;298:93–97. 31272739 10.1016/j.ijcard.2019.06.056

[8] Li J, Wang Y, Lin J, et al. Soluble CD40L is a useful marker to predict future strokes in patients with minor stroke and transient ischemic attack. Stroke. 2015;46(7):1990–1992. 26012640 10.1161/STROKEAHA.115.008685

[9] Berthet C, Lei H, Gruetter R, et al. Early predictive biomarkers for lesion after transient cerebral ischemia. Stroke. 2011;42(3):799–805. 21293024 10.1161/STROKEAHA.110.603647

[10] Li J, Wang Y. Blood biomarkers in minor stroke and transient ischemic attack. Neurosci Bull. 2016;32(5):463–468. 27250628 10.1007/s12264-016-0038-5 PMC5563758

[11] Guan L, Wang Y, Claydon VE, et al. Autonomic parameter and stress profile predict secondary ischemic events after transient ischemic attack or minor stroke. Stroke. 2019;50(8):2007–2015. 31238826 10.1161/STROKEAHA.118.022844

[12] Kim J, Kim YD, Song TJ, et al. Red blood cell distribution width is associated with poor clinical outcome in acute cerebral infarction. Thromb Haemost. 2012;108(08):349–356. 22739700 10.1160/TH12-03-0165

[13] Wang L, Wang C, Wu S, et al. Red blood cell distribution width is associated with mortality after acute ischemic stroke: a cohort study and systematic review. Ann Transl Med. 2020;8(4):81. 32175374 10.21037/atm.2019.12.142 PMC7049007

[14] Zhao Z, Liu T, Li J, et al. Elevated red cell distribution width level is associated with oxidative stress and inflammation in a canine model of rapid atrial pacing. Int J Cardiol. 2014;174(1):174–176. 24750719 10.1016/j.ijcard.2014.03.189

[15] Cherubini A, Ruggiero C, Polidori MC, et al. Potential markers of oxidative stress in stroke. Free Radic Biol Med. 2005;39(7):841–852. 16140205 10.1016/j.freeradbiomed.2005.06.025

[16] Chamorro A, Dirnagl U, Urra X, et al. Neuroprotection in acute stroke: targeting excitotoxicity, oxidative and nitrosative stress, and inflammation. Lancet Neurol. 2016;15(8):869–881. 27180033 10.1016/S1474-4422(16)00114-9

[17] Amarenco P. Transient ischemic Attack. N Engl J Med. 2020;382(20):1933–1941. 32402163 10.1056/NEJMcp1908837

[18] Goldstein LB, Jones MR, Matchar DB, et al. Improving the reliability of stroke subgroup classification using the trial of ORG 10172 in acute stroke treatment (TOAST) criteria. Stroke. 2001;32(5):1091–1098. 11340215 10.1161/01.str.32.5.1091

[19] Clarke K, Sagunarthy R, Kansal S. RDW as an additional marker in inflammatory bowel disease/undifferentiated colitis. Dig Dis Sci. 2008;53(9):2521–2523. 18259864 10.1007/s10620-007-0176-8

[20] Xie KH, Liu LL, Su CY, et al. Low antioxidant status of serum uric acid, bilirubin, albumin, and creatinine in patients with benign paroxysmal positional vertigo. Front Neurol. 2020;11:601695. 33329359 10.3389/fneur.2020.601695 PMC7714964

[21] Lappegard J, Ellingsen TS, Skjelbakken T, et al. Red cell distribution width is associated with future risk of incident stroke. The Tromsø Study. Thromb Haemost. 2016;115(1):126–134. 26290352 10.1160/TH15-03-0234

[22] Hong RH, Zhu J, Li ZZ, et al. Red blood cell distribution width is associated with neuronal damage in acute ischemic stroke. Aging. 2020;12(10):9855–9867. 32445553 10.18632/aging.103250 PMC7288978

[23] Wang C, Wang L, Zhong D, et al. Association between red blood cell distribution width and hemorrhagic transformation in acute ischemic stroke patients. Cerebrovasc Dis. 2019;48(3–6):193–199. 31786566 10.1159/000504742

[24] Pinho J, Marques SA, Freitas E, et al. Red cell distribution width as a predictor of 1-year survival in ischemic stroke patients treated with intravenous thrombolysis. Thromb Res. 2018;164:4–8. 29438871 10.1016/j.thromres.2018.02.002

[25] Saliba W, Barnett-Griness O, Elias M, et al. The association between red cell distribution width and stroke in patients with atrial fibrillation. Am J Med. 2015;128:192–198. 10.1016/j.amjmed.2014.09.020 25447618

[26] Peng YF, Pan GG. Red blood cell distribution width predicts homocysteine levels in adult population without vitamin B12 and folate deficiencies. Int J Cardiol. 2017;227:8–10. 27846467 10.1016/j.ijcard.2016.11.012

[27] Lorente L, Martin MM, Abreu-Gonzalez P, et al. Early mortality of brain infarction patients and red blood cell distribution width. Brain Sci. 2020;10(4):196. 10.3390/brainsci10040196 PMC7226572 32224967

[28] Semba RD, Patel KV, Ferrucci L, et al. Serum antioxidants and inflammation predict red cell distribution width in older women: the women’s health and aging study I. Clin Nutr. 2010;29(5):600–604. 20334961 10.1016/j.clnu.2010.03.001 PMC3243048

[29] Wang X, Lo EH. Triggers and mediators of hemorrhagic transformation in cerebral ischemia. Mol Neurobiol. 2003;28(3):229–244. 14709787 10.1385/MN:28:3:229

[30] Wang J, Zhang P, Tang Z. Animal models of transient ischemic attack: a review. Acta Neurol Belg. 2020;120(2):267–275. 32048230 10.1007/s13760-020-01295-5 PMC7083805

[31] Warner DS, Sheng H, Batinić-Haberle I. Oxidants, antioxidants and the ischemic brain. J Exp Biol. 2004;207(18):3221–3231. 15299043 10.1242/jeb.01022

[32] McCormick MT, Muir KW, Gray CS, et al. Management of hyperglycemia in acute stroke: how, when, and for whom? Stroke. 2008;39(7):2177–2185. 18436889 10.1161/STROKEAHA.107.496646

[33] Arnao V, Salemi G, Scondotto S, et al. Stroke incidence and case fatality: a 9-year prospective population-based study in an elderly population of bagheria, Italy. Neurol Sci. 2021;42(6):2447–2452. 33078249 10.1007/s10072-020-04830-7 PMC8159798

[34] Hollander M, Koudstaal PJ, Bots ML, et al. Incidence, risk, and case fatality of first ever stroke in the elderly population. The Rotterdam Study. J Neurol Neurosurg Psychiatry. 2003;74(3):317–321. 12588915 10.1136/jnnp.74.3.317 PMC1738313

[35] Rosenzweig S, Carmichael ST. Age-dependent exacerbation of white matter stroke outcomes: a role for oxidative damage and inflammatory mediators. Stroke. 2013;44(9):2579–2586. 23868277 10.1161/STROKEAHA.113.001796 PMC3791618

[36] Patel KV, Ferrucci L, Ershler WB, et al. Red blood cell distribution width and the risk of death in middle-aged and older adults. Arch Intern Med. 2009;169(5):515–523. 19273783 10.1001/archinternmed.2009.11 PMC2765040

[37] Stoll G, Bendszus M. Inflammation and atherosclerosis: novel insights into plaque formation and destabilization. Stroke. 2006;37(7):1923–1932. 16741184 10.1161/01.STR.0000226901.34927.10

[38] van der Spuy WJ, Pretorius E. Interrelation between inflammation, thrombosis, and neuroprotection in cerebral ischemia. Rev Neurosci. 2012;23(3):269–278. 22752784 10.1515/revneuro-2012-0028

[39] Anrather J, Iadecola C. Inflammation and stroke: an overview. Neurotherapeutics. 2016;13(4):661–670. 27730544 10.1007/s13311-016-0483-x PMC5081118

[40] Ramiro L, Simats A, Garcia-Berrocoso T, et al. Inflammatory molecules might become both biomarkers and therapeutic targets for stroke management. Ther Adv Neurol Disord. 2018;11:1756286418789340. 30093920 10.1177/1756286418789340 PMC6080077

[41] Segal HC, Burgess AI, Poole DL, et al. Population-based study of blood biomarkers in prediction of subacute recurrent stroke. Stroke. 2014;45(10):2912–2917. 25158774 10.1161/STROKEAHA.114.005592 PMC5380212

[42] Ansari FA, Ali SN, Mahmood R. Sodium nitrite-induced oxidative stress causes membrane damage, protein oxidation, lipid peroxidation and alters major metabolic pathways in human erythrocytes. Toxicol in Vitro. 2015;29(7):1878–1886. 26231821 10.1016/j.tiv.2015.07.022

[43] Pierce CN, Larson DF. Inflammatory cytokine inhibition of erythropoiesis in patients implanted with a mechanical circulatory assist device. Perfusion. 2005;20(2):83–90. 15918445 10.1191/0267659105pf793oa

[44] Lippi G, Targher G, Montagnana M, et al. Relation between red blood cell distribution width and inflammatory biomarkers in a large cohort of unselected outpatients. Arch Pathol Lab Med. 2009;133(4):628–632. 19391664 10.5858/133.4.628

[45] Ahmad H, Khan M, Laugle M, et al. Red cell distribution width is positively correlated with atherosclerotic cardiovascular disease 10-Year risk score, age, and CRP in spondyloarthritis with axial or peripheral disease. Int J Rheumatol. 2018;2018:2476239. 30363719 10.1155/2018/2476239 PMC6181003

[46] de Gonzalo-Calvo D, de Luxan-Delgado B, Rodriguez-Gonzalez S, et al. Interleukin 6, soluble tumor necrosis factor receptor I and red blood cell distribution width as biological markers of functional dependence in an elderly population: a translational approach. Cytokine. 2012;58(2):193–198. 22309694 10.1016/j.cyto.2012.01.005

[47] Crimi E, Ignarro LJ, Napoli C. Microcirculation and oxidative stress. Free Radic Res. 2007;41(12):1364–1375. 18075839 10.1080/10715760701732830

[48] Kaul DK, Koshkaryev A, Artmann G, et al. Additive effect of red blood cell rigidity and adherence to endothelial cells in inducing vascular resistance. Am J Physiol Heart Circ Physiol. 2008;295(4):H1788–H1793. 18757485 10.1152/ajpheart.253.2008

[49] Tikhomirova IA, Oslyakova AO, Mikhailova SG. Microcirculation and blood rheology in patients with cerebrovascular disorders. Clin Hemorheol Microcirc. 2011;49(1–4):295–305. 22214701 10.3233/CH-2011-1480

[50] Yedgar S, Koshkaryev A, Barshtein G. The red blood cell in vascular occlusion. Pathophysiol Haemost Thromb. 2002;32(5–6):263–268. 13679654 10.1159/000073578

[51] Hong L, Fang K, Ling Y, et al. Red blood cell distribution width is associated with collateral flow and final infarct volume in acute stroke with large artery atherosclerosis. Semin Thromb Hemost. 2020;46(4):502–506. 31858514 10.1055/s-0039-3400257

[52] Lappegard J, Ellingsen TS, Vik A, et al. Red cell distribution width and carotid atherosclerosis progression. The Tromsø Study. Thromb Haemost. 2015;113(3):649–654. 25631329 10.1160/TH14-07-0606

