# Supplementary material for: Red cell distribution width: a novel predictive biomarker for stroke risk after transient ischaemic attack
Source: Ann Med. 2022 Apr 26;54(1):1167–77. doi: 10.1080/07853890.2022.2059558 (PMC9045760; doi:10.1080/07853890.2022.2059558)
Supplement: Supplemental Material [file IANN_A_2059558_SM6537.doc]

**TIA medical history acquisition Scale**

**Patient name: Sex: Age: [P](../../../../C:/Program%20Files%20(x86)/Youdao/Dict/8.10.5.0/resultui/html/index.html#/javascript:;)hone:**

**[A](../../../../C:/Program%20Files%20(x86)/Youdao/Dict/8.10.5.0/resultui/html/index.html#/javascript:;)ddress: [H](../../../../C:/Program%20Files%20(x86)/Youdao/Dict/8.10.5.0/resultui/html/index.html#/javascript:;)eight：  CM   [W](../../../../C:/Program%20Files%20(x86)/Youdao/Dict/8.10.5.0/resultui/html/index.html#/javascript:;)eight：  kg**

**Time of [history-taking](../../../../C:/Program%20Files%20(x86)/Youdao/Dict/8.10.5.0/resultui/html/index.html#/javascript:;): Examiner: Date:**

**Reliability of history sure□ basically sure□ not sure□**

**Time of TIA onset:**

**[S](../../../../C:/Program%20Files%20(x86)/Youdao/Dict/8.10.5.0/resultui/html/index.html#/javascript:;)ymptom: Duration of symptoms:**

**Number of episodes up to the time of visit:**

**Positive signs :**

**NIHSS score: ABCD^2^ score: MRS score:**

**Medication history:**

**Stroke type: LAA□ SVO□ CE□ SUE□**

**Head CT results：**

**Head MRI+DWI+MRA results ：**

**Transcranial Doppler：**

**Carotid color duplex ultrasonography：**

**Dynamic electrocardiogram：**

**History of stroke: Hypertension: Diabetes mellitus: Coronary heart disease: Alcohol consumption: Current smoking:**

**[F](../../../../C:/Program%20Files%20(x86)/Youdao/Dict/8.10.5.0/resultui/html/index.html#/javascript:;)amily medical history:**
